# Citations—be sure to have a good title

**DOI:** 10.1093/jhps/hnx025

**Published:** 2017-06-01

**Authors:** Richard (Ricky) Villar

**Affiliations:** Editor-in-Chief, Journal of Hip Preservation Surgery

There is a sad feature about scientific publishing; not all papers published are ever cited. Can you imagine, spending 2 years undertaking a study and then no one ever acknowledges your effort? Estimates vary but some citation analyses suggest that 90% of academic papers are never cited and 50% are never read by anyone other than the authors, reviewers and a journal’s publication team [1]. Such a finding clearly brings into question the impact factor, as a journal’s impact can be influenced by relatively few papers. To have your paper published in a high-impact-factor journal does not, alas, mean that your own paper contributed to that high impact.

In addition, does a high citation rate mean a decent level of evidence? Several orthopaedic subspecialties have looked at this. In elbow surgery, e.g. the 50 topmost cited papers were published between 1950 and 2010, the number of citations ranging from 124 to 388 and the most common level of evidence was Level IV [2]. The same has been done for distal radial fractures, where the topmost 100 cited articles were published between 1951 and 2009, citations ranged from 67 to 525 and again, the majority were Level IV [3]. For hip surgery, we also do not fare well, with the top 100 papers published between 1945 and 2013 contributing between 290 and 3144 citations. However, only 1% of the citation classics was a randomized controlled trial (RCT) [4]. Orthopaedic cartilage surgery does better as its topmost 50 cited articles were published between 1968 and 2008, citations ranged from 172 to 989 but significantly for cartilage research, stronger levels of evidence led to increased citations [5].

As an Editor-in-Chief, more than occasionally one sees submissions that are judged badly by reviewers but which still make it somehow into print. Papers that were once rejected can at times be highly cited. So, what is it that attracts the eye of the researcher-cum-reader and leads to a work being widely read? Is it all to do with content and the scientific value of the research? Not always, if the figures I quote are believed. How does a paper work its way up the citation pile, akin to being on the first page of Google? Perhaps I should start with something simple. How about the title?

The title plays a key part in encouraging a paper to be cited. Just think of when you last entered a proper bookshop. Musty wooden shelves, tables scattered around, laden with books by authors of whom the public may never have heard. How many of us have opportunity-purchased a book based purely on title and cover design? I will wager you have. If not then I bet it crossed your mind.

Titles do make a difference. The title that is most predictive of success, certainly within the ecological literature, and there is no reason why hip preservation should be any different, is a title that emphasizes broader conceptual or comparative issues [6]. The more specific the title the less likely it is that you will be cited. So often authors make their titles long, burdensome and specific because they feel it adds something deeply academic to their work. Far from it. Never forget that readers are human. They scan read, have busy lives and are subject to the same influences as the rest of mankind. Remember that wander through the bookshop and what it was that caught your eye? I will wager it was the title. Once a reader is hooked, a citation may be on its way.

There is a difference, too, between what might influence a reviewer, for that matter an editor, and what might encourage a researcher to cite. It appears that intermediate length titles are more successful during editorial review and papers with subtitles are less likely to be rejected. However, neither of these features is predictive of citations.

The medical educators have looked at this as well [7], recognizing that the title of a paper offers a crucial portal into any scientific field. It is the first thing a browser sees, the trigger that might one day increase the impact. They found that the mean length of title in medical education peaked in the 2000s, dropping to 70 characters in the 2010s, with no titles being longer than 140 characters (the length of a Tweet) in the last decade. Titles posed as a question have increased steadily and have now settled at 11%. Humour has also begun to be used suggesting that even in this digital era, authors realize that papers tend to be selected by humans, not by machine. I can certainly vouch for that with *JHPS*. The title may not be everything but it is certainly hugely important. However, do be extremely careful about using humour.

Turning to the last issue of our Journal, your Journal, our utterly magnificent *JHPS*, what is there to say? A massive amount, of course, far more than the journal has space to publish. The review by Viswanath and Khanduja [8] on whether hip arthroscopy can delay the need for hip arthroplasty is a question that has long been on many surgeons’ minds. I thought the review was brilliant. If you have not looked at it, please do so. I swear it will be useful. I was also taken by the paper from Byrd and Jones [9] on the arthroscopic repair of torn hip abductors, perhaps influenced by sustaining exactly that injury myself and then spending a month hobbling, wondering which of my colleagues I might trust to do the necessary deed. I was upset to learn that to sustain a hip abductor tear I should really be a female in my mid-50s—I am not. I have decided to carry on hobbling.

And in this issue, issue 4.2? Once again, I am spoilt for choice. However, leaping out at me is the paper by Mardones *et al.* [10] from Santiago, Chile, who report on a small study of mesenchymal stem cell injections into osteoarthritic hips. Clearly, there is more work to be done but it is good to see that their patients improved, some of whom were followed for as long as 30 months. Those presently undertaking orthobiological therapies will be aware that this is presently an area of intense focus worldwide and appears to be under regular attack. How often have we seen such things when new procedures start to develop, frequently with laboratory researchers and clinicians at illogical loggerheads?

Another paper I was pleased to read was that by Kraeutler *et al.* [11] looking at return to running after hip arthroscopic surgery, just about the commonest question my patients ask before their operation. The paper also suggests a rehabilitation protocol and the final sentence of their Abstract says all, “There is an overall lack of published outcomes based on patients adhering to various post-hip arthroscopy rehabilitation protocols”. This is certainly an area we should all look at in more detail.

So, as ever, please enjoy this issue of *JHPS*. It is published for you, the hip preservation practitioner, and is filled from cover to cover with pearls. I commend this issue to you in its entirety.

My very best wishes to you all.
